# miR-98 Modulates Cytokine Production from Human PBMCs in Systemic Lupus Erythematosus by Targeting IL-6 mRNA

**DOI:** 10.1155/2019/9827574

**Published:** 2019-12-01

**Authors:** Shiwen Yuan, Chun Tang, Dongying Chen, Fangfei Li, Mingcheng Huang, Jinghua Ye, Zhixiang He, Weinian Li, Yi Chen, Xiaojun Lin, Xiaodong Wang, Xiaoyan Cai

**Affiliations:** ^1^Department of Rheumatology, Guangzhou First People's Hospital, School of Medicine, South China University of Technology, No 1, Panfu Road, Guangzhou, China; ^2^Department of Nephrology, Kidney and Urology Center, The Seventh Affiliated Hospital of Sun Yat-sen University, Shenzhen, China; ^3^Department of Rheumatology, The First Affiliated Hospital of Sun Yat-sen University, No 58, Zhongshan 2nd Road, Guangzhou, China; ^4^Department of Ultrasound, The First Affiliated Hospital of Guangzhou University of Chinese Medicine, No 16, Jichang Road, Guangzhou, China

## Abstract

**Objective:**

There is evidence that interleukin-6 (IL-6) upregulation plays a critical role in immunopathology of systemic lupus erythematosus (SLE). MicroRNA- (miRNA-) 98 was predicted to bind with the 3′-untranslated region (3′-UTR) of IL-6 gene. We hypothesized miR-98 through its regulation of IL-6 gene expression to influence cytokine production from peripheral blood mononuclear cells (PBMCs) in SLE.

**Methods:**

The expression of miR-98 and IL-6 mRNA in the PBMCs of 41 SLE patients and 20 healthy controls (HC) was detected by quantitative reverse transcription PCR (qRT-PCR). The correlations between miR-98 expression and clinical features were evaluated. Luciferase reporter assay was performed to identify miR-98 targets. miR-98 mimics, miR-98 inhibitor, and IL-6 overexpression vector were generated. Cell viability of PBMCs was assessed using MTT assay. Gene expression and protein level were determined by qRT-PCR and Western blotting. TNF-*α*, IL-8, IL-1*β*, and IL-10 levels in cultured supernatants were quantified using ELISA.

**Results:**

The expression of miR-98 was downregulated in PBMCs of SLE patients, and its expression is negatively associated with IL-6 levels. miR-98 expression was correlated with disease activity, lupus nephritis, and anti-dsDNA antibody. IL-6 mRNA was a target gene of miR-98. IL-6 overexpression promoted the proliferation of PBMCs and increased the levels of TNF-*α*, IL-8, IL-1*β*, and IL-10. Those effects were further enhanced by miR-98 inhibitor, while were suppressed by miR-98 mimics. miR-98 regulated the levels of STAT3 phosphorylation via its target gene IL-6.

**Conclusion:**

The current study revealed that miR-98 could ameliorate STAT3-mediated cell proliferation and inflammatory cytokine production via its target gene IL-6 in patients with SLE. These results suggest that miR-98 might serve as a potential target for SLE treatment and other IL-6-mediated diseases.

## 1. Introduction

Systemic lupus erythematosus (SLE) is a chronic and incurable autoimmune disease with a breakdown of self-tolerance that leads to various immune abnormalities, including the production of autoantibodies to double-stranded DNA and other nuclear antigens and accumulation of immune complexes in the kidney and other important organs [1]. Until now, the etiology and pathogenesis of SLE are not well clarified. Genetic, epigenetic, cytokine, hormonal, and environmental factors might all be involved in the initial and development of SLE [2].

Cytokines are believed to play important roles in modulating the immune response against foreign or self-antigen [3, 4]. A number of studies have identified that many cytokine levels including interleukin- (IL-) 6, IL-10, and tumor necrosis factor- (TNF-) *α* are significantly elevated in SLE [5, 6]. IL-6 is a proinflammatory cytokine produced by antigen-presenting cells. Data from several studies suggest that elevated levels of IL-6 are implicated in regulating disease activity and in the involvement of different organs in patients with SLE [7, 8]. However, the mechanisms governing the regulation of cytokines in SLE remain elusive.

MicroRNAs (miRNAs) are single stranded, small short noncoding RNA strands, usually 22 nucleotides in length, ubiquitously expressed in human cells and tissues [9]. During the last few years, it has become clear that miRNAs participate in numerous physiological and pathological processes. miRNAs regulate gene expression at the posttranscriptional level. Numerous studies have shown that miRNAs are critical for the development and function of the immune system [10–13]. However, the functional role of miRNAs in cytokines regulating in patients with SLE has not been previously investigated.

In the present study, we predicted specific miRNAs which could bind with the 3′ untranslated region (3′UTR) of IL-6 mRNA using the online software TargetScan (http://www.targetscan.org/vert_71/) and identified that miR-98 indeed targeted IL-6. Based on these findings, we aimed to investigate the expression and function of miR-98, especially its potential role in regulating cytokines in SLE.

## 2. Materials and Methods

### 2.1. Patients and Controls

Forty-one SLE patients classified according to the 1997 American College of Rheumatology (ACR) criteria for SLE [14] were recruited from Guangzhou First People's Hospital from March to May 2017. Twenty age- and sex-matched healthy controls (HC) from the same general population were recruited voluntarily. In the SLE group, there were 37 females and 4 males; the mean age was 34.1 ± 16.6 years. In the control group, there were 14 females and 6 males; the mean age was 32.6 ± 14.1 years. All the control samples were collected from the physical examination center. Approvals were obtained from the Ethics Committee of Guangzhou First People's Hospital and the Ethics Committee of Jinan University based on the ethical guidelines of the 2008 Declaration of Helsinki, and informed consent was obtained from all study participants.

Clinical and demographic information was collected from admission records, including gender, age, serological examinations, organ involvement, lupus disease activity, and therapeutic medications. Laboratory test results included erythrocyte sedimentation rate (ESR), C-reactive protein (CRP), compliment 3, immunoglobulin G (IgG), serum creatinine (SCr), serum albumin (ALB), anti-cardiolipin antibody (aCL), anti-*β*2-glycoprotein 1 (*β*2-GP1) antibody, antinuclear antibody (ANA), anti-double-stranded DNA (ds-DNA) antibody, anti-Sm antibody, anti-RNP antibody, and anti-Ro/La antibody. Organs involved included skin, joints, serosae, renal, and central nervous system. Lupus nephritis (LN) was defined if clinical and laboratory manifestations met the ACR criteria [15]. Central nervous system involvement consisted of 12 manifestations of neuropsychiatry syndromes which were defined in ACR [16]. Disease activity was assessed on the basis of the SLE Disease Activity Index (SLEDAI) [17]. Medications including corticosteroids and additional immunosuppressive agents were also recorded.

### 2.2. Cell Isolation and Cell Culture

Human peripheral blood mononuclear cells (PBMCs) of SLE patients and HC were isolated from heparinized blood by density gradient centrifugation. After isolation, PBMCs were cultured in RPMI 1640 medium supplemented with 10% fetal calf serum (FCS) and penicillin/streptomycin in U-bottom 24-well plates. The amount of cells/mL was adjusted at a density of 2 × 10^6^ cells/well.

### 2.3. Plasmid Construction

A STAT3 promoter was cloned and inserted into pGL3-control vector using XhoI and MluI to construct pGL3-STAT3. An IL-6 ORF was cloned and inserted into pcDNA3.1 vector using HindIII and EcoRI to construct pcDNA3.1-IL6. Plasmids were transfected into PBMCs using Lipofectamine 2000.

### 2.4. MTT Assay

PBMC viability was assessed using an MTT assay according to the manufacturer's instructions. Briefly, PBMCs were transfected with miR-98 mimics, miR-98 inhibitor, IL-6 overexpression vector, and/or treated with S31-201 in a 96-well plate for 24 h. MTT reagent (0.5 mg/mL) was then added to each well. After a 4 h incubation at 37°C, the formazan crystals were dissolved in DMSO and the absorbance was recorded at 570 nm using a microplate reader (ThermoFisher).

### 2.5. RNA Isolation and Real-Time Quantitative RT-PCR

PBMC total RNA was isolated using the TRIzol reagents (Invitrogen, San Diego, CA, USA). cDNA was synthesized from 2 *μ*g of total RNA using a reverse transcription kit from (ThermoFisher, MA, USA). TNF-*α*, IL-8, IL-1*β*, and IL-10 mRNA levels were determined by real-time PCR using SYBR Green mix (Takara, Dalian, Liaoning, China). Primers are listed in Table [Supplementary-material supplementary-material-1]. RT of miR-98 was performed from 10 ng of total RNA using All-in-One™ miRNA qPCR Primer (HmiRQP0853, GeneCopoeia, Guangzhou, China) and the All-in-One™ miRNA qRT-PCR Detection Kit (QP015, GeneCopoeia, Guangzhou, China). cDNA obtained from this step was used for quantitative TaqMan PCR using the real-time primers provided, according to the manufacturer's instructions. Cq values were converted to fold expression changes (2^-*ΔΔ*Cq^ values) following normalization to U6 small nuclear RNA. For mRNA analysis, RT was performed on total RNA using random primers (Promega), and *β*-actin was used to control for cDNA concentration in a separate PCR reaction for each sample. 2^-*ΔΔ*Cq^ values were used to calculate the expression of each mRNA.

### 2.6. Luciferase Reporter Assay

A luciferase reporter assay was conducted using HEK293T cells. After 48 h transfection with miR-98 mimics or inhibitor, 800 ng pcDNA3.1 vector or pcDNA3.1-IL6 plasmids, along with 1 ng pRL-TK and 200 ng pGL3-STAT3 plasmid, HEK293T cells were harvested for luciferase activity assessment using a dual-luciferase reporter assay system (Promega). The final results were normalized to Renilla luciferase activity. The results are representative of at least three independent experiments.

### 2.7. Enzyme-Linked Immunosorbent Assay (ELISA)

TNF-*α* (DTA00C), IL-8 (D8000C), IL-1*β* (QLB00B), and IL-10 (D1000B) levels in cultured supernatants were quantified using an ELISA kit (R&D Systems, Minneapolis, MN, USA) according to the manufacturer's instructions. Assays were performed in triplicate.

### 2.8. Western Blotting

PBMC proteins were extracted using RIPA lysis buffer with a proteinase inhibitor. The protein concentration in the lysates was measured by the BCA protein assay kit (#23227, Pierce, ThermoFisher), and 50 *μ*g of the total protein mixed with 4x SDS loading buffer was loaded per lane. The proteins in the lysates were separated by 12% SDS-PAGE and transferred to polyvinylidene difluoride (PVDF) membranes (EMD Millipore, Billerica, MA, USA). In order to block nonspecific binding, the PVDF membranes were incubated with 5% skim milk powder at room temperature for 1 h. The PVDF membranes were then incubated for 12 h at 4°C with an antiserum containing antibodies against IL-6 (#12912, Cell Signaling Technology), Stat3 (#9139, Cell Signaling Technology), Phospho-Stat3 (Tyr705) (#9145, Cell Signaling Technology), and GAPDH (MA5-15738, Invitrogen). Primary was diluted at 1 : 1,000. A peroxidase-conjugated secondary antibody (1 : 5,000 dilution, BOSTER Biological Technology Co. Ltd, Wuhan, China) and enhanced chemiluminescence western blot detection reagents (NCI4106, Pierce, ThermoFisher) were used to visualize the target proteins, which were quantified with ChemiDoc XRS+ (Bio-Rad Laboratories, Inc.).

### 2.9. Statistical Analyses

The differences of continuous variables with nonnormal distribution and ordered categorical variables between two groups were compared by the Mann-Whitney *U* test. Student's *t* test was used to compare the differences of continuous variables with normal distribution, and chi-square for categorical variables. Mean ± SD or median and interquartile range was presented for continuous or ordinal data. Categorical variables were presented as the absolute count and percentage. Statistical analyses were performed using the SPSS 21.0 package. A *p* value less than 0.05 was considered to be statistically significant.

## 3. Results

### 3.1. The Expression of miR-98 Is Decreased in SLE PBMCs

**Figure 1:**
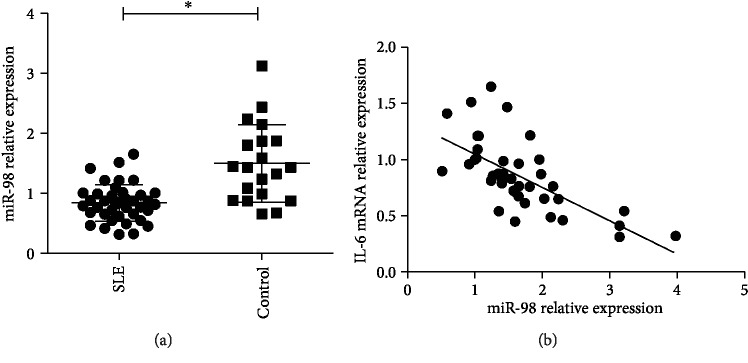
miR-98 expression is decreased in SLE. (a) The expression of miR-98 was detected in PBMCs of 41 SLE patients and 20 healthy controls using qRT-PCR; ^∗^*p* < 0.05. (b) The correlation between the expression of IL-6 mRNA and expression of miR-98 in PBMCs of SLE patients was analyzed by two-tailed Pearson's correlation analysis, *r* = −0.695; *p* < 0.001.

The expression of endogenous miR-98 in PBMCs of 41 SLE patients and 20 HC was detected by qRT-PCR. The results showed that the expression of miR-98 was much lower in SLE PBMCs compared to that in HC PBMCs (*p* < 0.05) (Figure 1(a)). miR-98 levels were presented as mean and standard deviation (SD). In this study, miR-98 low expression was considered when the expression level of miR-98 was below or equal to mean-SD from HC PBMCs, miR-98 high expression was considered when the expression level of miR-98 was above or equal to mean + SD from HC PBMCs, and miR-98 normal expression was considered when the expression level of miR-98 was ranging from mean-SD to mean + SD. It was noted that the ratio of miR-98 low expression samples in the SLE group was significantly higher than that in the control group (Table 1), suggesting an underlying association between decreased miRNA expression and pathogenesis of SLE.

### 3.2. miR-98 Expression Is Negatively Correlated with IL-6 Levels and miR-98 Low Expression Was Correlated with LN

The correlations between miR-98 expression and clinical features were evaluated. Compared with patients without miR-98 low expression, patients with miR-98 low expression showed significantly higher rates of LN and arthritis. There was no relationship between miR-98 expression and gender, age, central nervous system involvement, or skin rash (Table 2). With regard to the laboratory data, miR-98 low expression was found to be correlated with anti-dsDNA antibody. Other laboratory data were not significantly different between these patients (Table 2). Patients with miR-98 low expression had a significantly higher level of SLEDAI score than that without miR-98 low expression. Furthermore, a significant negative correlation was observed between miR-98 expression and IL-6 mRNA expression in SLE PBMCs (*r* = −0.695, *p* < 0.001) (Figure 1(b)).

### 3.3. miR-98 Directly Targets the 3′-UTR of IL-6 mRNA

**Figure 2:**
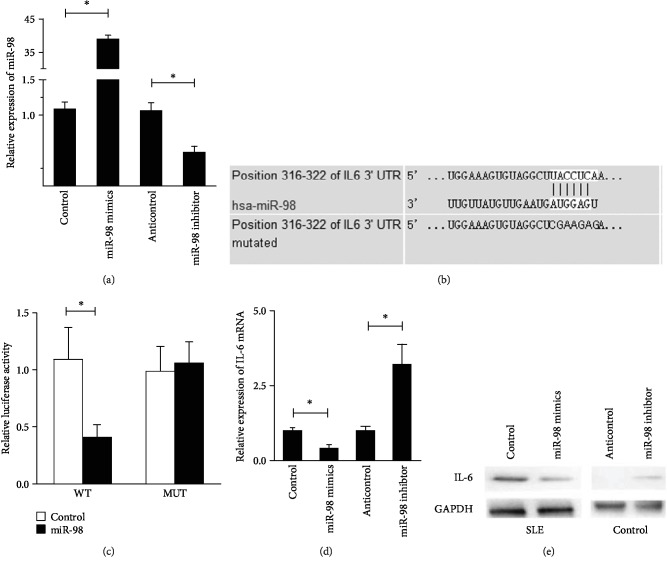
IL-6 is a target of miR-98 in SLE. (a) PBMCs of SLE patients were transfected with miR-98 mimics, inhibitor, or control sequences, then miR-98 levels were detected by qRT-PCR. U6 was used as an internal control. (b) The predicted miR-98 binding site within IL-6 mRNA 3′UTR and its mutated version by site mutagenesis are as shown. (c) Luciferase assay was performed in healthy PBMCs that were cotransfected with miRNA mimics or control sequences and reporter vectors carrying IL-6 mRNA 3′UTR with wild type (pMIR-IL-6-WT) versus mutated type (pMIR-IL-6-MUT), respectively. (d) PBMCs of SLE patients were transfected with miR-98 mimics or with unrelated sequences as controls, then PBMCs of SLE patients were transfected with anticontrol or miR-98 inhibitors, respectively. (e) Western blotting was used to test IL-6 expression; GAPDH was used as an internal control. Data are means of three separated experiments ± SD; ^∗^*p* < 0.05.

Eukaryotic expression vectors of miR-98 mimics and miR-98 inhibitor were generated. The transfection efficacies of miR-98 mimics and inhibitor in PBMCs were detected and verified by qRT-PCR. Compared with the control vector (control) and anticontrol vector (anticontrol) transfected PBMCs, miR-98 production was increased in the miR-98 mimics group and decreased in the miR-98 inhibitor group (Figure 2(a)). By means of TargetScan6.0 online software, IL-6 was found to be a putative target of miR-98 (Figure 2(b)). We hypothesized miR-98 by binding IL-6 mRNA to perform its biological functions.

A dual-luciferase reporter assay was performed to demonstrate a direct interaction between IL-6 and miR-98 in healthy PBMCs. The group with cotransfected miR-98 mimics with luciferase vectors bearing WT IL-6 mRNA 3′-UTR target sequences showed the lowest luciferase activity. The luciferase activity was not significantly different between cotransfected control and miR-98 mimics with luciferase vectors bearing the mutated IL-6 mRNA 3′-UTR group (Figure 2(c)), suggesting that miR-98 is indeed binding to 3′UTR of IL-6 mRNA.

The effects of miR-98 on IL-6 production by SLE PBMCs using both qRT-PCR and Western blot analysis were evaluated. We found that IL-6 expression was significantly suppressed in the miR-98 mimics group at mRNA and protein levels, whereas it was boosted in the miR-98 inhibitor group (Figures 2(d) and 2(e)), indicating that the expression of IL-6 is negatively regulated by miR-98.

### 3.4. miR-98 Downregulates IL-6-Mediated PBMC Proliferation and Inflammatory Cytokine Production in SLE

**Figure 3:**
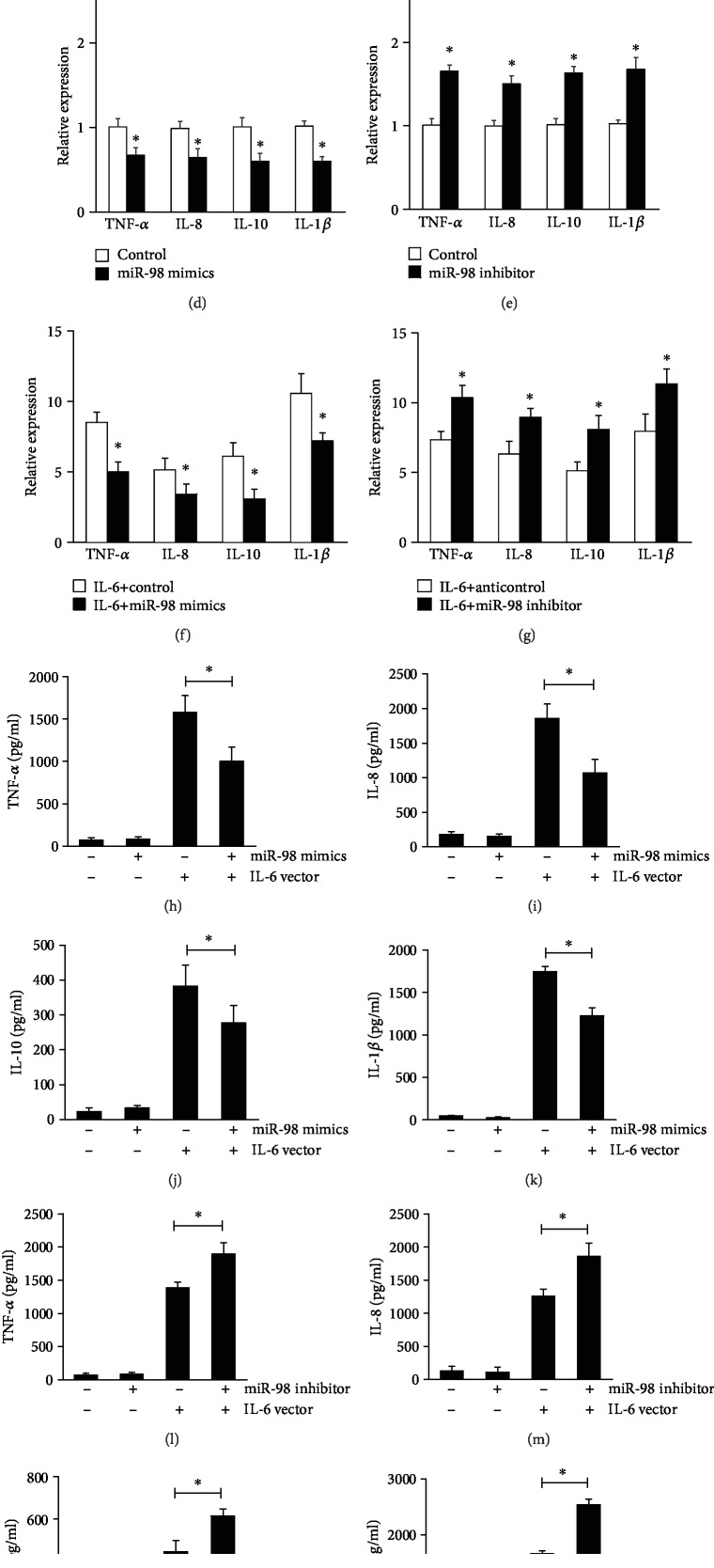
IL-6 reverses miR-98-mediated PBMC function in SLE. (a) PBMC cells of SLE patients were transfected with IL-6 overexpression vector or empty vector, then IL-6 protein levels in supernatants were detected by ELISA. MTT assay shows that overexpression of IL-6 could disrupt the inhibition effect of miR-98 mimics on PBMCs of SLE patients (b) and inhibition of IL-6 could enhance the effect of miR-98 inhibitor on PBMCs (c). PBMCs were transfected with miR-98 mimics and/or IL-6 overexpression vector; the expression of TNF-*α*, IL-8, IL-1*β*, and IL-10 in cells and their levels in a medium was detected by qRT-PCR (d, e) and ELISA (h–k), respectively. PBMCs were transfected with miR-98 inhibitor and/or IL-6 overexpression vector; the expression of TNF-*α*, IL-8, IL-1*β*, and IL-10 in cells and their levels in a medium was detected by qRT-PCR (f, g) and ELISA (l–o), respectively. Data are means of three separated experiments ± SD; ^∗^*p* < 0.01.

To further investigate the function of miR-98 and whether the effects of miR-98 were mediated through IL-6, we transfected control vector or IL-6 overexpression vector into SLE PBMCs with miR-98 mimics or miR-98 inhibitor. The levels of IL-6 overexpression vector in SLE PBMCs were measured by ELISA (Figure 3(a)). MTT assay was used to measure the effect of miR-98 on the proliferation of SLE PBMCs. Proliferation of PBMCs was analyzed at 48 h. The results indicated that miR-98 mimics suppressed the proliferation of PBMCs and that overexpression of IL-6 reversed this inhibition (Figure 3(b)), while miR-98 inhibitor increased the proliferation of SLE PBMCs and was further enhanced by IL-6 overexpression (Figure 3(c)).

In addition, we found that the levels of TNF-*α*, IL-8, IL-1*β*, and IL-10 in SLE PBMCs were markedly downregulated by miR-98 mimic transfection (Figure 3(d)) and that were markedly upregulated when miR-98 was suppressed (Figure 3(e)). IL-6 overexpression increased the levels of TNF-*α*, IL-8, IL-1*β*, and IL-10, which were further enhanced in the miR-98 inhibitor group but were suppressed in the miR-98 mimics group (Figures 3(f)–3(o)). These results indicated that miR-98 could ameliorate IL-6-mediated cell proliferation and inflammatory cytokine production in patients with SLE.

### 3.5. miR-98 Regulates STAT3 Phosphorylation Level and STAT3-Mediated PBMC Proliferation and Inflammatory Cytokine Production via IL-6 in SLE

**Figure 4:**
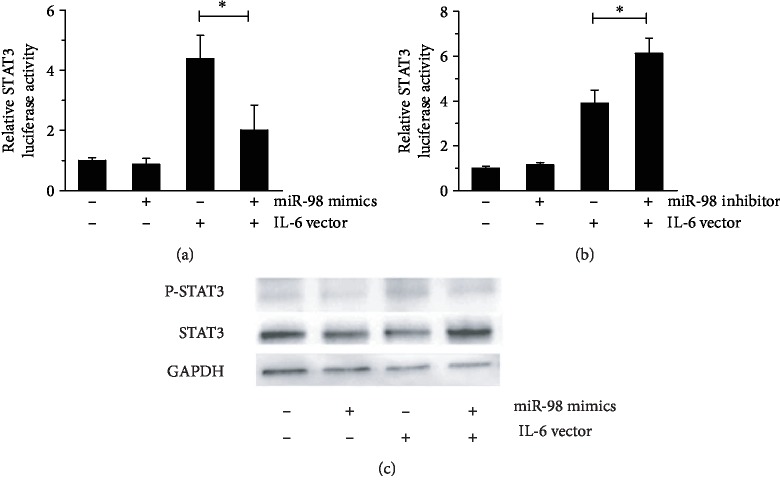
miR-98 can upregulate STAT3 levels in the presence of IL-6. HEK293T cells were transfected with pRL-TK, pGL3-STAT3, miR-98 mimics/inhibitor, and/or IL-6 overexpression vector; the activity of STAT3 was analyzed by luciferase reporter assay (a, b); the protein expression of STAT3 and phosphorylated STAT3 was detected by Western blotting (c). Data are means of three separated experiments ± SD; ^∗^*p* < 0.01.

Signal transducer and activator of transcription 3 (STAT3) protein plays a central role in transmitting IL-6 signals. Our data showed that either miR-98 mimics or miR-98 inhibitor could not alter the luciferase activity of STAT3 (Figures 4(a) and 4(b)) and could not regulate the phosphorylation of STAT3 (Figure 4(c)). However, when the STAT3 activity was stimulated by IL-6, the miR-98 mimics could suppress IL-6-mediated STAT3 activation, and miR-98 inhibitor led to an opposite effect (Figures 4(a) and 4(b)). In addition, it was noted that the level of phosphorylated STAT3 was lower when IL-6 vector was cotransfected with miR-98 mimics than IL-6 vector transfection alone (Figure 4(c)), suggesting that binding to the IL-6 mRNA 3′-UTR is crucial for the regulation of STAT3 phosphorylation by miR-98.

**Figure 5:**
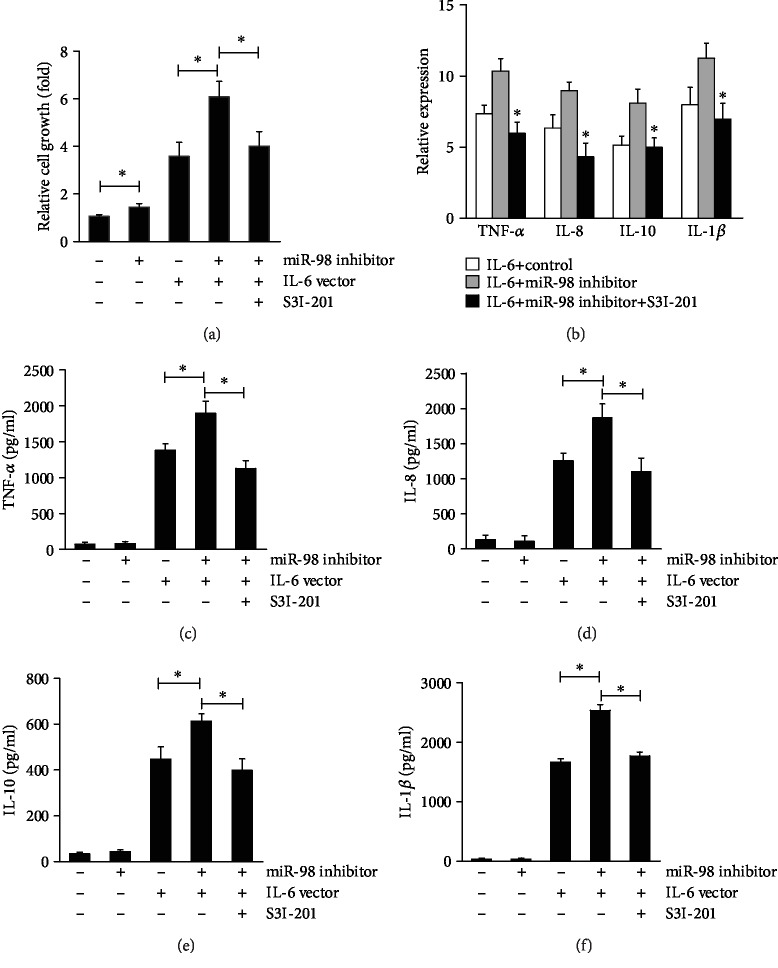
STAT3 regulates IL-6-mediated miR-98 functions in SLE PBMCs. PBMCs from SLE patients were transfected with miR-98 inhibitor or NC, IL-6 overexpression vector or empty vector, and/or S3I-201; cell growth was analyzed by MTT assay (a); the expression of TNF-*α*, IL-8, IL-1*β*, and IL-10 in cells was detected by qRT-PCR (b); the levels of TNF-*α*, IL-8, IL-1*β*, and IL-10 in a medium was tested by ELISA (c, d). Data are means of three separated experiments ± SD; ^∗^*p* < 0.01.

Our data showed that either suppression of miR-98 or overexpression of IL-6 could promote PBMC proliferation, while STAT3 inhibitor S31-201 could abrogate the PBMC proliferative effect stimulated by miR-98 inhibitor cotransfected with IL-6 vector (Figure 5(a)). The levels of TNF-*α*, IL-8, IL-1*β*, and IL-10 from PBMCs were increased by miR-98 inhibitor combined with IL-6 vector transfection (Figure 5(b)), while STAT3 inhibitor S31-201 could reverse those effects (Figures 5(c)–5(f)). These results indicated that miR-98 could inhibit IL-6-mediated cell proliferation and inflammatory cytokine production via STAT3 in patients with SLE.

## 4. Discussion

Recent development in genetics and epigenetics has improved our understanding of SLE pathogenesis. Identifying differentially expressed genes and the mechanisms that regulate them will provide a comprehensive understanding of the initiation and development of SLE. Zhu et al. performed whole-genome transcription analysis using PBMCs from 30 SLE patients, including 15 with LN and 15 without LN, and 25 normal controls. They identified 552 upregulated genes and 550 downregulated genes in PBMCs of SLE, and of special note, they found IL-6 to be upregulated [18]. Our previous study had also shown that IL-6 was both upregulated and hypomethylated in SLE PBMCs [19].

In the current study, our aim was to investigate the regulatory mechanism of IL-6 in SLE. miRNAs can serve as novel biomarkers for SLE, both as useful diagnostic and prognostic tools [10]. TargetScan software predicted that miRNAs might bind to the 3′UTR of IL-6 mRNA. This prediction suggested that miR-98 was a potential regulator for the expression of IL-6. We found that miR-98 was significantly downregulated in SLE PBMCs relative to controls. miR-98 has been shown to be dysregulated in many different diseases. As noted by previous literature, miR-98 was downregulated in tumors, including non-small-cell lung cancer [20], leukemia [21], hepatocellular carcinoma [22], and breast cancer [23]. Chen et al. reported that the expression of miR-98 was upregulated in B cells isolated from mouse hearts with myocarditis [24]. In our study, the expression of miR-98 was remarkably lower in SLE PBMCs compared to that in HC PBMCs. We then examined the relationship between miR-98 levels and clinical parameters. The results indicated that low expression of miR-98 was correlated with disease activity, renal involvement, and anti-dsDNA antibody. Anti-dsDNA antibody was one of the hallmarks of the disease in terms of diagnosis and an important biomarker of SLE activity and kidney involvement or dysfunction [25, 26]. Our results indicated that miR-98 might be correlated with disease phenotype and serve as a new disease activity biomarker in SLE.

A significant negative correlation was observed between miR-98 expression and IL-6 mRNA expression in SLE PBMCs. Our study demonstrated that miR-98 was indeed binding to 3′UTR of IL-6 mRNA and the expression of IL-6 is negatively regulated by miR-98. IL-6 mRNA has been identified as a target of miR-98 in other diseases and tissues in previously reported experiments. Li et al. was the first to identify IL-6 mRNA as a target of miR-98 in melanoma [27]. Ji et al. identified that miR-98 was significantly downregulated in nucleus pulposus (NP) tissues in patients with intervertebral disc degeneration relative to controls. They found that miR-98 significantly promoted type II collagen expression in NP cells and that knockdown of IL-6 induced effects on NP cells similar to those induced by miR-98 [28]. Another study demonstrated that monocyte chemotactic protein-1 induced IL-6 expression in THP-1 macrophage cells via downregulating miR-98 [29].

The imbalance of proinflammatory cytokines, such as IL-6, TNF-*α*, IL-1*β*, and IL-10, was demonstrated that contributed to immune dysfunction and also mediated inflammation of the tissues and organ damage in SLE [30]. We further examined the effect of miR-98 on functional activity in PBMCs. We found that miR-98 inhibition could promote the proliferation of PBMCs, and inhibition of miR-98 combined with overexpression of IL-6 could increase the levels of TNF-*α*, IL-8, IL-1*β*, and IL-10 in PBMCs but were suppressed by miR-98 mimics. These results indicated that miR-98 could ameliorate IL-6-mediated cell proliferation and inflammatory cytokine production in patients with SLE. This finding suggested that enhancing the expression of miR-98 in SLE might have clinical benefits.

STAT3 protein plays a central role in transmitting cytokine signals. STAT3 signaling was identified to play a key role in suppressing TNF-*α* synthesis by human monocytes in the course of systemic inflammation in vivo. IL-10 levels were enhanced in the serum and tissues of patients with SLE. STAT3 and recruitment to the IL-10 promoter had been demonstrated to induce IL-10 expression in SLE [5]. In this study, we identified that miR-98 could not regulate the expression and activation of STAT3 unless IL-6 was present. Furthermore, we found that the STAT3 inhibitor S31-201 could abrogate the combined effects of miR-98 inhibitor plus IL-6 overexpression vector. Therefore, we speculated that miR-98 might suppress PBMC cytokine production via IL-6/STAT3.

The current study systematically analyzed the expression and function of miR-98 in SLE PBMCs. However, it had its own limitations as an experiment in vitro from a heterogeneous cell population (PBMCs). Further research investigating the function of miR-98 from each PBMC subpopulations in SLE is required. Moreover, additional investigation on transgenic animal models would help us to identify the in vivo function of miR-98.

In conclusion, the expression of miR-98 is downregulated in SLE PBMCs. miR-98 might be correlated with disease phenotype and serve as a new disease activity biomarker in SLE. miR-98 downregulation contributes to IL-6-mediated PBMC proliferation and inflammatory cytokine production in SLE. miR-98 regulates the STAT3 phosphorylation level via IL-6 in SLE. miR-98 might serve as a potential target for SLE treatment.

## Figures and Tables

**Table 1:** Comparison of miR-98 expression in PBMCs between SLE patients and healthy controls (HC).

	miR-98 expression		*χ* ^2^	*p*
	Low expression	Normal and overexpression		
SLE	21	20	9.564	0.002
HC	2	18		

**Table 2:** Comparison of demographic and clinical characteristics between SLE patients with and without miR-98 low expression.

	Patients with miR-98 low expression (*n* = 21)	Patients without miR-98 low expression^∗^ (*n* = 20)	*p*
Female, *n* (%)	17 (81.0)	20 (100.0)	0.126
Age at SLE diagnosis, yrs, mean ± SD	34.27 ± 20.25	30.54 ± 7.37	0.606
Skin rash, *n* (%)	6 (28.6)	10 (50.0)	0.160
Oral ulcer, *n* (%)	7 (33.3)	9 (45.0)	0.401
Arthritis, *n* (%)^∗∗^	16 (28.6)	10 (50.0)	0.001
Polyserositis, *n* (%)	4 (19.0)	3 (15.0)	0.732
Lupus nephritis, *n* (%)^∗∗^	14(66.7)	6 (30.0)	0.019
Central nervous system involvement, *n* (%)	4 (19.0)	2 (10.0)	0.706
SCr (*μ*mol/L), mean ± SD	121.80 ± 81.19	98.46 ± 63.64	0.093
ALB (g/L), mean ± SD	26.71 ± 6.32	28.42 ± 5.25	0.120
Complement 3 (g/L), mean ± SD	0.37 ± 0.14	0.44 ± 0.26	0.133
IgG (g/L), mean ± SD	12.72 ± 5.63	16.01 ± 6.68	0.154
ACL (IgM/IgG), *n* (%)	1 (4.8)	1 (5.0)	0.938
Anti-*β*2-GP1 (IgG), *n* (%)	2 (9.5)	1 (5.0)	0.720
ANA, *n* (%)	19 (90.5)	19 (95.0)	0.593
Anti-ds-DNA, *n* (%)^∗∗^	13 (61.9)	3 (15.0)	0.004
Anti-Sm, *n* (%)	4 (19.0)	0 (0.0)	0.107
Anti-Ro, *n* (%)	12 (57.1)	7 (35.0)	0.105
Anti-La, *n* (%)	3 (14.3)	1 (5.0)	0.606
Anti-U1RNP, *n* (%)	4 (19.0)	2 (10.0)	0.663
Corticosteroid therapy, *n* (%)	21 (100.0)	19 (95.0)	0.773
IV CYC therapy, *n* (%)	14 (66.7)	9(45.0)	0.577
MMF therapy, *n* (%)	3 (14.3)	2 (10.0)	0.682
Hydroxychloroquine therapy, *n* (%)	19 (90.5)	19 (95.0)	0.593
Disease activity (SLEDAI, mean ± SD)^∗∗^	14.93 ± 7.91	7.13 ± 6.68	0.032

^∗^Patients with normal and overexpression of miR-98; ^∗∗^*p* value < 0.5.

## Data Availability

The experimental and clinical data used to support the findings of this study are included within the supplementary information file.

## Supplementary Materials

Supplementary Materials The provided supplementary material is the primers for qRT-PCR in this study.
