# Supplementary material for: miR-98 Modulates Cytokine Production from Human PBMCs in Systemic Lupus Erythematosus by Targeting IL-6 mRNA
Source: J Immunol Res. 2019 Dec 1;2019:9827574. doi: 10.1155/2019/9827574 (PMC6914974; doi:10.1155/2019/9827574)
Supplement: Supplementary Materials — The provided supplementary material is the primers for qRT-PCR in this study. [file 9827574.f1.pdf]

Table S1 Primers for qRT-PCR

	right primer (5’-3’)	left primer (5’-3’)
IL-6	CAAATTCGGTACATCCTC	CTGGCTTGTTCCTCACTA
TNF-α	CACGCTCTTCTGCCTGCT	GCTTGTCACTCGGGGTTC
IL-8	AGTGAAGATGCCAGTG	TTCTAGCAAACCCATT
IL-10	TGCCTTCAGCAGAGTG	GAGGGTCTTCAGGTTC
IL-1β	TATTACAGTGGCAATGAGG	GATGAAGGGAAAGAAGGT
β-actin	GGGCACGAAGGCTCATCATT	AGCGAGCATCCCCCAAAGTT
U6	CTCGCTTCGGCAGCACA	AACGCTTCACGAATTTGCGT
miR-98 mimics	UGAGGUAGUAAGUUGUAUUGUU	
mi-NC	UUCUCCGAACGUGUCACGUTT	
